# Evaluation of the recombinant antigens B2t and 2B2t, compared with hydatid fluid, in IgG-ELISA and immunostrips for the diagnosis and follow up of CE patients

**DOI:** 10.1371/journal.pntd.0006741

**Published:** 2018-09-06

**Authors:** Ana Hernández-González, Carlos Sánchez-Ovejero, Raúl Manzano-Román, María González Sánchez, José Manuel Delgado, Teresa Pardo-García, Francisco Soriano-Gálvez, Okan Akhan, Carmen M. Cretu, Kamenna Vutova, Francesca Tamarozzi, Mara Mariconti, Enrico Brunetti, Ambra Vola, Massimo Fabiani, Adriano Casulli, Mar Siles-Lucas

**Affiliations:** 1 Instituto de Salud Carlos III, Centro Nacional de Microbiología, Majadahonda, Madrid, Spain; 2 Instituto de Recursos Naturales y Agrobiología de Salamanca (IRNASA-CSIC), Salamanca, Spain; 3 Vircell SL, Parque Tecnológico de la Salud, Granada, Spain; 4 Faculty of Medicine, Hacettepe University, Ankara, Turkey; 5 University of Medicine and Pharmacy, Colentina Clinical Hospital - Parasitology, Bucharest, Romania; 6 Specialised Hospital of Infectious and Parasitic Diseases "Prof. Ivan Kirov", Department of Infectious, Parasitic and Tropical Diseases, Medical University, Sofia, Bulgaria; 7 Centre for Tropical Diseases, Sacro Cuore-Don Calabria Hospital, Negrar, Verona, Italy; 8 Department of Clinical Surgical Diagnostic and Paediatric Sciences, University of Pavia, Pavia, Italy; 9 Department of Clinical Surgical Diagnostic and Paediatric Sciences, University of Pavia, and Division of Infectious and Tropical Diseases, San Matteo Hospital Foundation, Pavia, Italy; 10 San Matteo Hospital Foundation, Pavia, Italy; 11 Infectious Diseases Department, Istituto Superiore di Sanità, Rome, Italy; 12 WHO Collaborating Centre for the epidemiology, detection and control of cystic and alveolar echinococcosis, Istituto Superiore di Sanità, Rome, Italy; 13 European Reference Laboratory for Parasites (EURLP), Istituto Superiore di Sanità, Rome, Italy; Consejo Nacional de Investigaciones Cientificas y Tecnicas, Fundación Mundo Sano, ARGENTINA

## Abstract

Cystic echinococcosis (CE) is one of the most widespread helminthic zoonoses and is caused by the tapeworm *Echinococcus granulosus* complex. CE diagnosis and monitoring primarily rely on imaging techniques, complemented by serology. This is usually approached by the detection of IgG antibodies against hydatid fluid (HF), but the use of this heterogeneous antigenic mixture results in a variable percentage of false positive and negative results, and has shown to be useless for follow-up due to the long persistence of anti-HF antibodies in cured patients. To improve test performances and standardization, a number of recombinant antigens mainly derived from HF have been described, among them the B2t and 2B2t antigens. The performance of these antigens in the diagnosis and follow up of patients with CE has been so far evaluated on a limited number of samples. Here, we evaluated the performances of tests based on B2t and 2B2t recombinant antigens compared to HF in IgG-ELISA and immunochromatography (IC) for the diagnosis and follow-up of patients with CE in a retrospective cohort study. A total of 721 serum samples were collected: 587 from 253 patients with CE diagnosed by ultrasonography (US), 42 from patients with alveolar echinococcosis and 92 from healthy donors from Salamanca (Spain). The highest overall sensitivity was obtained with HF in ELISA (85.5%), followed by IC containing HF and 2B2t-HF (83.0% and 78.2%, respectively). The lowest sensitivity was obtained with B2t and 2B2t in ELISA (51.8%). The highest specificity was obtained with IC containing 2B2t-HF (100%), and the lowest with HF-ELISA (78.0%). The lowest cross-reactivity with sera from patients with alveolar echinococcosis was detected with the recombinant antigens in ELISA (9.5% - 16.7%) and the highest with the HF-IC (64.3%). The results of B2t and 2B2t-ELISA were influenced by cyst stage, as classified by US according to the WHO-Informal Working Group on Echinococcosis (WHO-IWGE), with low sensitivity for inactive (CE4 and CE5) cysts, and by the drug treatment, with higher sensitivity in patients after drug treatment compared with patients not subjected to drug treatment. The two recombinant antigens in ELISA provided promising results for monitoring patients in follow-up, although their use is limited to patients with positive serology against them at the beginning of the follow-up. Potential biological reasons behind the low sensitivity of the recombinant antigens and possible strategies to enhance the performance of CE serology are discussed.

## Author summary

Cystic echinococcosis (CE) is a helminthic zoonosis caused by *Echinococcus granulosus* sensu lato. CE diagnosis and monitoring is of paramount importance for the clinical management of patients and primarily rely on imaging techniques, complemented by serology. CE serology is usually based on the detection of antibodies against hydatid fluid (HF), but the use of this heterogeneous antigenic mixture shows several drawbacks, including false positive and negative results, unsatisfied predictive values, and long persistence of detectable antibody levels in cured patients. As an alternative, to improve test performances and standardization, several recombinant antigens have been described, but these have been so far evaluated only on a limited number of samples. Here, two recombinant antigens derived from one of the immunodominant HF antigens (antigen B2) have been tested in enzyme-linked immunosorbent assay (ELISA) and in immunochromatographic strips (IC) against 721 serum samples. Although more specific than the HF, the recombinant antigens in ELISA showed low sensitivity for patients with inactive (CE4 and CE5) cysts and for patients not subjected to drug treatment. This limited their use for follow-up, although promising, to those patients with positive serology at the beginning of the follow-up period. These results will aid in the future development of a serological test with enhanced performance in the diagnosis and follow-up of patients with CE.

## Introduction

Cystic echinococcosis (CE) is a widespread zoonosis caused by *Echinococcus granulosus sensu lato*. Human CE is clinically complex and difficult to manage, due to the growth of the parasitic cysts in different organs (although liver involvement represents around 70% of cases; [1]) with variable clinical outcomes. Currently the management of CE encompasses surgery, percutaneous drainage, pharmacological treatment with benzimidazoles, and the “watch and wait” approach [2]. Clinical management is mainly based on cyst stage according to the WHO Informal Working Group on Echinococcosis (WHO-IWGE) applicable to liver cysts, ultrasound cyst classification and other clinical factors [2, 3].

The diagnosis of CE is primarily based on imaging, complemented by serology. However, the performance of current serodiagnostic tools based on the detection of antibodies against hydatid fluid (HF) is far from optimal, due to poor specificity, sensitivity, and tests standardization. Factors influencing the sensitivity of serodiagnostic tests include, among others, cyst number, size and stage, and timing of serum collection in correlation to treatment (rev. in [4]; [5, 6]). A second point of concern in using HF-based tests is their low specificity, as sera from patients with a wide range of other infectious and non-infectious diseases, some of which commonly found in CE endemic areas (e.g. alveolar echinococcosis–AE-, cysticercosis, clonorchiasis, fasciolosis, schistosomosis, ascariosis, amoebosis and malignancy) have been described as cross-reacting with this crude antigen (rev. in [4]; [5, 7, 8]). Especially high (>50%) is the cross-reactivity of HF with sera from patients with AE [9]. False-positive reactions against HF have been also described in healthy donors, with especially low specificity in areas where CE is endemic (e.g. specificity of 53.8% in Iranian donors; [10]). Although this could be partially attributed to contact with parasite eggs, at the moment this occurrence could not be verified, its frequency is not quantified, and, in any case, apparently it does not anticipate the future development of a CE cyst [11] and is therefore useless for clinical purposes. Additionally, antigens used for the serological diagnosis of CE are not standardized, partly accounting for the large variability of results when applied in different settings, and the difficulty in comparing results from different groups. Finally, HF-based serology tests are of limited use for follow-up due to the long persistence of anti-HF antibodies even in cured patients [12]. Although monitoring of titres over time can indicate the outcome of therapy, this is not always clear-cut and years long follow-up of patients with imaging is required to monitor the evolution of cysts. Relapse rates vary greatly between reports, depending on cyst localization, stage, and type of therapy. In case of treatment of active hepatic cysts, mean relapse rates range from 2% to 24% after surgery [13, 14], to ≤15% after percutaneous treatment [15, 16, 17], to an average of 25% at 2 years after albendazole therapy [18, 19]. Clearly, the possibility to differentiate with a serological marker between patients with good progression (to inactive cysts) and cured patients from patients with recurrences is key, to provide a more precise prognosis and shorten patient follow-up [20, 21].

Some recombinant antigens have shown better potential than HF to differentiate CE from other pathologies by serology and for the follow-up of treated patients, including several isoforms of the antigen B (AgB1, B2, B3 and B4; e.g., [22, 23]) and the antigen 5 (Ag5), among others (rev. in [4]; [5]). AgB and Ag5 are the most abundant immune reactive molecules identified in HF [24]. Both antigens are expressed in all parasite’s life cycle stages, and AgB isoforms, encoded by a multigene family having at least five gene loci subunits (B1–B5) appear to be variably expressed among *E*. *granulosus* strains, life cycle stages and cysts stages [25, 26]. In previous publications, our group described the recombinant antigens B2t and 2B2t, derivatives of antigen B2 (AgB2) and representing one and two tandem–head to tail- repeats of the *E*. *granulosus* G1 genotype AgB2 lacking the signal peptide, respectively, which showed preliminary good sensitivity and specificity in IgG-ELISA and good potential for the follow-up of patients with CE after surgical treatment [9, 12]. Later, we showed that after surgical treatment, antibodies to the recombinant antigens 2B2t and EgP29 became negative in the majority of CE-confirmed, surgically cured patients included in the study. Nevertheless, the relatively low percentage of patients positive to these recombinant antigens before treatment raised doubts about their use for documenting primary cure [27]. However, these studies were limited by the low number of patients included and the deficiency of some patient’s clinical data.

The achievement of a reliable serological diagnosis of CE could serve as an adjunctive to image techniques, especially when a challenge in distinguishing between an echinococcal cyst and other lesions, ranging from simple cysts to neoplasms, is found, and in follow-up cases when the interpretation of the treatment outcome is difficult (e.g. in distinguishing between post-surgical cavity and relapse).

The development of easy-to-use diagnostic devices based on the use of recombinant antigens is also a much-needed development in CE, to provide accessible testing in underserved areas where equipped laboratories are lacking, and to support field studies [28]. Five recent studies evaluated the performances of some of the currently commercially available rapid diagnostic tests for the diagnosis of CE [28–32] (rev. in [4, 33]). The performances of these rapid diagnostic tests seem overall comparable to those of traditional lab-based assays, however, as for other serodiagnostic tests, rapid diagnostic tests still lack standardization and show unsatisfactory sensitivity and specificity.

To further assess the performance of B2t and 2B2t recombinant antigens in comparison to HF, we evaluated the three antigen preparations in IgG-ELISA on an extended panel of sera from patients with CE, both for the primary diagnosis of CE and for the follow-up of patients after different clinical management approaches. Additionally, the 2B2t antigen was used to develop a rapid immunochromatographic test containing the recombinant antigen in the test line and compared with the commercial kit VIRapid HYDATIDOSIS based on a semi-purified fraction of HF enriched in Ag5 and AgB (Vircell S. L., Granada, Spain) for its diagnostic and monitoring performances.

## Materials and methods

### Study design and samples

A retrospective cohort study was conducted among patients with CE identified and followed-up by ultrasonography (US; reference standard) at the Division of Infectious and Tropical Diseases, San Matteo Hospital Foundation, Pavia, Italy, from 1998 to 2010. Number of patients in this cohort were 253. CE cysts detected in these patients were classified according to the recommendations of the WHO-IWGE on liver cysts [3], with minor modifications regarding cyst activity [34], as follows: active cysts CE1, CE2 and CE3b, transitional cysts CE3a, and inactive cysts CE4 and CE5. Patients with more than one cyst were classified according to the stage of the most active cyst.

For the follow-up analysis, all patients with at least two consecutive sera were considered. Changes in cyst stage during the follow-up period were assessed by US. Patients who had undergone surgery or an aspiration technique were divided into “cured patients”, if showing no US images suggesting relapses during the follow-up period, and “non-cured patients”, if showing relapses detected by US during the monitoring period. Patients treated with albendazole were divided into patients with good response when the image evolved from active or transitional stages to inactive stages (CE4 and CE5) and with poor response when the image did not change from active or transitional stages to inactive stages. Patients without treatment, included in the “watch and wait” approach (showing CE4 or CE5 cysts) were also checked for US stage changes during the follow-up period.

To check the specificity and cross-reactivity of the serological tests, 42 samples from patients with AE confirmed by Em2plus serology were donated by Prof. Gottstein (Institute of Parasitology, University of Berne, Switzerland), and 92 sera from healthy donors were kindly donated by Dr. Muñoz (University Hospital, Salamanca, Spain).

Human samples used in this study are included in the EchinoBiobank.

### Ethics statement

The use and transfer of stocked human serum leftover from routine analyses carried out in San Matteo Hospital Foundation, Pavia, Italy, was approved by the Ethics Committee of IRCCS San Matteo Hospital Foundation, Pavia, Italy (Acceptance Report 2015041 of 06/07/2015). All samples used in this study were anonymized.

### Antigens

HF was aseptically obtained from fertile sheep hydatid cysts at the Coreses slaughterhouse (Zamora, Spain) from sheep processed as part of the normal work of the abattoir and after receiving consent, using sterile syringes and gauges. The HF was centrifuged at 1,000 g for 5 min, and the protein concentration in the supernatant measured with the Micro BCA Protein Assay Kit (Pierce). The supernatant was stored at -80°C until used.

The expression vectors pGEX-4T2 and pGEX-4T1 (GE Healthcare) containing the relevant nucleotide sequences of the antigens B2t and 2B2t were used to transform *Escherichia coli* BL21 CodonPlus RIL competent cells (Stratagene). Induction, expression, purification and thrombin cleavage of both proteins were performed as previously described [9, 12].

Antigens used in this study are available in the EchinoBiobank (https://biobancos.isciii.es/ListadoColecciones.aspx; collection nb. C0003432), a collection of human and animal samples constituted under the umbrella of the HERACLES FP7 project [35] (http://www.heracles-fp7.eu/).

### Test methods

Ninety-six-well polystyrene plates (Corning, Spain) were incubated at 4°C overnight with 100 μl/well of HF (5 μg/ml), B2t or 2B2t (0.5 μg/ml) in carbonate buffer (pH 9.6). Plates were then washed six times with phosphate-buffered saline (PBS) (pH 7.4) with 0.05% Tween 20 (washing buffer) and blocked for 1.5 h at 37°C with 200 μl 1% bovine serum albumin (BSA; Sigma Aldrich, Spain) in washing buffer. Sera were then added in duplicate (100 μl/well) at 1:200 dilution in blocking buffer, and plates incubated for 1 h at 37°C. After washing as described above, the secondary antibody (peroxidase-labeled rabbit anti-human IgG; Sigma Aldrich, Spain) was added (100 μl/well) at a 1:2,000 dilution in blocking buffer, and plates incubated for 1 h at 37°C. After one further washing as described above, the reaction was developed with 100 μl/well of citrate buffer (pH 5), plus orthophenylene diamine (0.28 mg/ml; Sigma Aldrich, Spain) and hydrogen peroxidase (0.4 μl/ml; Sigma Aldrich, Spain). The reaction was stopped with 50 μl/well of 3N sulfuric acid (Panreac, Spain), and plates were read at 492 nm in an ELISA reader (EAR 400; SLT Lab Instruments, Germany).

The serological index (SI) was calculated for each optical density in each plate using the following formula: [(NC-S)/(NC-PC)]x100, where NC and PC represent the negative and positive controls, respectively, and S stands for each serum. Negative control consisted of a pool of 10 sera from healthy donors with an OD in ELISA of 0.1 to 0.2 and positive control was a pool of 10 sera from patients with CE with an OD in ELISA of 0.4 to 0.5. The use of SI was elected to avoid biases in the OD due to assay variability.

**Fig 1:**
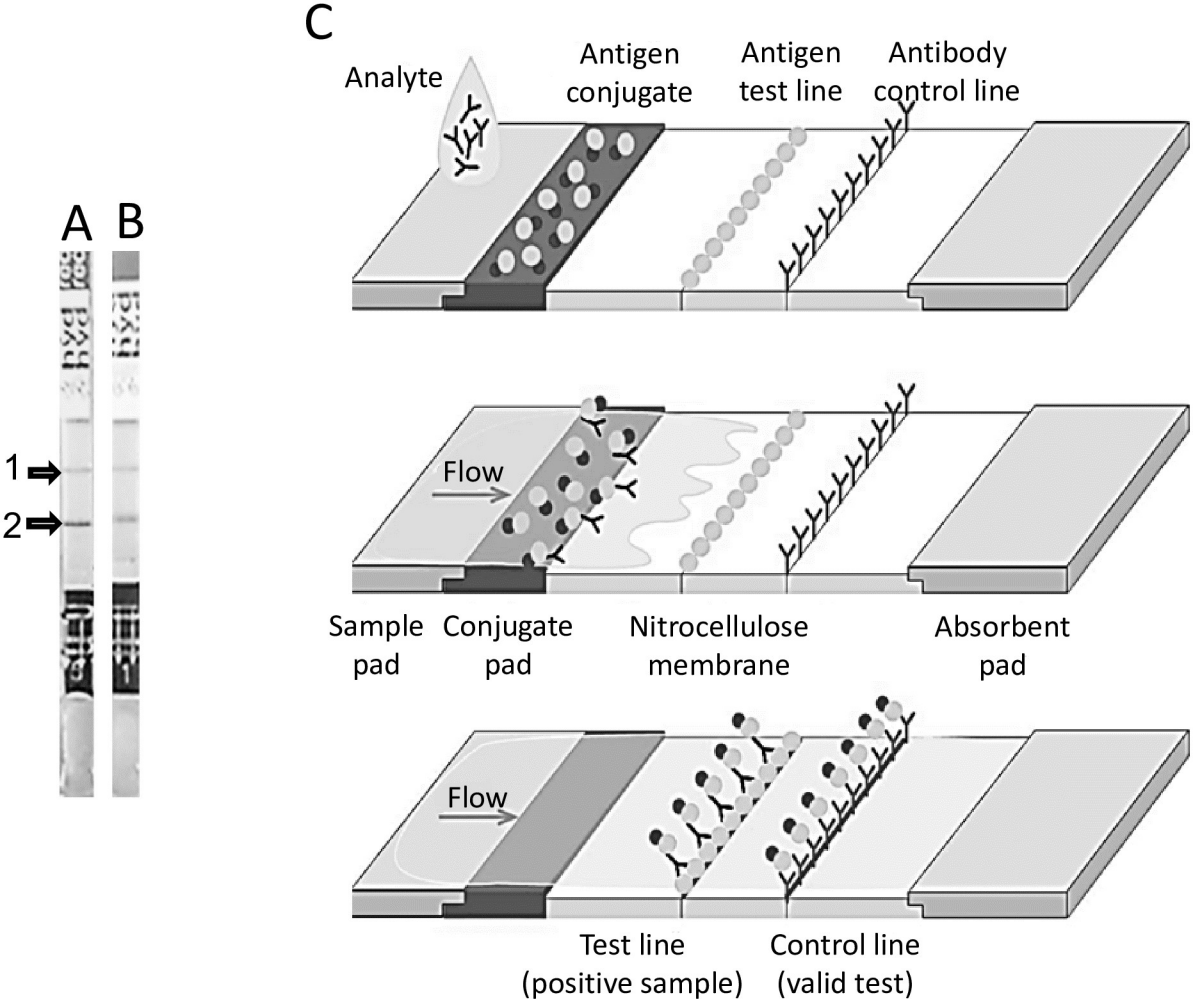
Immunochromatographic test containing the 2B2t recombinant antigen (A) or hydatid fluid (B) in the test line. Two positive tests are shown in the Fig 1, control line; 2, test line. C, schematic representation of the immunochromatograpic strip and its components.

An immunochromatographic strip (IC) carrying the recombinant 2B2t antigen in the test line and a semi-purified fraction of HF enriched in Ag5 and AgB (5/B) in the conjugate was developed by Vircell S.L. (Granada, Spain) (Fig 1). Briefly, the 2B2t recombinant antigen was dispensed in the test line (Biodot ZX1000, UK) on a nitrocellulose membrane (Millipore HF135, UK) at concentrations ranging from 0.5 to 5 mg/ml on different polystyrene cards (Lohmann, USA) diluted in PBS pH 7.2 (Sigma Aldrich, Spain) using 5% methanol (Panreac, Spain) as co-precipitator agent. Cards were dried for 10 minutes at 50°C (0–250 °C Indelab 6741 A, Spain). Chicken IgY (Jackson Immunoresearch, UK) at 0.3 mg/ml (PBS pH 7.2) was used as the control line. The 5/B antigen conjugate was prepared with 80 nm colloidal gold particles, manufactured by the seeding growth method defined by [36]. Protein conjugation was performed at 5 μg/ml in 5 mM phosphate buffer pH 7 for 15 minutes, followed by two blocking steps, with PEG 20,000 1% (Sigma Aldrich, Spain) and BSA 0.5% (ID Bio, France) respectively. After four centrifugation cycles at 17,500 rpm for 20 minutes (Sigma 3K30, Germany), the coated particles were resuspended in 5 mM sodium tetraborate buffer pH 8 (Sigma Aldrich, Spain), containing 0.5% BSA and 0.025% PEG 20,000. The conjugate for the control line of the test was prepared with the same particles and method aforementioned, and the adsorbed protein was donkey anti chicken IgY (Jackson Immunoresearch, UK). Both conjugates were diluted in drying buffer pH 8 (sodium tetraborate 5 mM, 1% BSA, 1% Tween 20 and 6% sucrose -Sigma Aldrich, Spain-) and then dried at 50°C on a 6 mm wide polyester pad–sample pad- (7403 grade; Hollingsworth & Vose, UK) for 20 minutes. The conjugate OD was determined in a UV-VIS spectrophotometer Cary 50 (Varian, Australia). The adsorbent pad was cotton-litter paper (470 grade; Whatman). All these elements, along with the 2B2t striped membrane, were assembled on a polystyrene card and then cut with an automatic guillotine (CM400; Biodot, UK). The different 2B2t concentrations in the test line were evaluated with serum samples already tested as positive or negative with the commercial Virapid Hydatidosis IC test [37], resulting 2.5 mg/ml the optimal concentration for the development of the reaction in the test line (Fig 1A).

The performance of this IC test was compared with that of the Virapid Hydatidosis commercial IC test from Vircell containing a semi-purified fraction of HF enriched in Ag5 and AgB in both the conjugate and the test line. After adding the sample (50 μl of serum) a colored band will appear in 10 min if the specific antibodies reacting with the antigen contained in the device are present in the sample. Each IC strip contains a control line for the validation of the assay.

IC tests were used according to the manufacturer’s instructions. Briefly, 30 μl of serum were applied in the sample well and 2 drops of developer solution were added to the well after 5 minutes. Results were read after 30 minutes. In order to perform the reading of the test and to determine the positivity of the samples, the intensity card included in the kit was used, indicating 4 levels of color intensity ranging from 0.5 to 3. When a visible test line has a color intensity lower than 0.5, the result is considered negative.

### Statistical analysis

For ELISA tests, the best cut-off value for each antigen (SI = 50) was previously determined by receiver operator characteristic analysis (ROC) using sera from patients with CE, considered true positive, and sera from donors plus sera from patients with AE, considered true negative [9]. Sera from patients with AE were considered true negative, due to the particular difficulties in differentiating some AE lesions from CE by imaging techniques, thus trying to define the less cross-reactive antigen in our settings.

Sensitivity, specificity and cross-reactivity of the three antigens in ELISA and IC were estimated together with 95% confidence interval (95% CI), and compared between paired samples through the McNemar test. Test sensitivity was assessed using the first time point serum available from each patient with CE, with the exclusion of patients visited for the first time after surgical or percutaneous treatment. Specificity was calculated as the percentage of negative reactions in serum samples from healthy donors, while cross-reactivity was calculated as the percentage of positive reactions in sera from patients with AE.

Sensitivity was also estimated separately for samples collected before and after albendazole treatment, and according to cysts characteristics (i.e., number, stage, size, and location). Differences in sensitivity by presence/absence of albendazole treatment and cyst characteristics were evaluated using multivariable logistic regression to account for potential confounding.

The follow-up analysis was conducted considering as baseline the first test available after surgical or percutaneous treatment, or after the time-point before the end of the last treatment cycle with albendazole. Time at first test available was considered for untreated patients.

The percentage of positive results of ELISA-based tests over time since the start of treatment (≤ 24 months; 25–48 months; > 48 months) was calculated and graphically compared between samples from cured and non-cured patients who underwent surgery/percutaneous treatment, and between samples from patients with good and poor response after drug treatment. Moreover, the same percentage over time since first testing was presented for samples from untreated patients.

Among patients who were positive at baseline, we compared rates of negativization between cured and not cured patients in those who underwent surgical or percutaneous treatment, and between patients with good and poor response in those treated with albendazole. Moreover, we compared rates of negativization among patients who underwent surgical or percutaneous treatment, those with a good response to albendazole treatment, and untreated patients. The incidence rates of negativization per 100 person-months (PM) of observation were estimated together with their 95% CI and differences among groups evaluated through the log-rank test. For each patient, person-time of observation was calculated as the number of months elapsed from baseline to last test available (patients who remained positive during follow-up), or from baseline to negativization (patients who became negative during follow-up). Time of negativization of ELISA was estimated by linear interpolation of SI values at the time of last positive test (SI ≥ 50) and time at first negative test (SI < 50), while time of negativization of IC was estimated as the mid-time between the last positive and first negative test.

Finally, we used the Wilcoxon rank-sum test to compare the median SI with interquartile range (IQR) in ELISA between patients treated with albendazole at the time they reached inactive cysts stage (CE4 or CE5) and untreated patients with inactive cysts in watch and wait at baseline.

P-values < 0.05 were considered statistically significant. All statistical analyses were performed using Stata 13.1 (StataCorp LP, College Station, Texas, USA).

## Results

### Samples

Demographics and relevant clinical data of the 253 patients with CE whose sera were included in the study are detailed in Table 1. All patients were older than 18 years. Presence of the most active cyst in locations other than liver were found in 18 patients, including peritoneum (6), spleen (4), lung (2), kidney (2), retrovesical (1), diaphragm (1), pelvic (1) and thigh (1).

**Table 1:** Demographic and clinical characteristics of 253 patients with CE.

Characteristics	Number (percentage)
**Gender**	
Male	136 (53.7%)
Female	117 (46.3%)
**Number of cysts**	
0*	33 (13%)
1	142 (56%)
>1	78 (31%)
**Cyst localization**	
Liver	231 (91.3%)
Others	18 (7.1%)
NS	4 (1.6%)
**Main cyst diameter**	
S (0–50 mm)	97 (38.3%)
M (>50–100 mm)	108 (42.7%)
L (>100 mm)	16 (6.3%)
NS	32 (12.7%)
**Cyst classification**	
CE1	13 (5.1%)
CE2	20 (7.9%)
CE3a	28 (11.1%)
CE3b	71 (28.1%)
CE4	49 (19.4%)
CE5	39 (15.4%)
NS	33 (13%)

S, small; M, medium; L, large.

*Patients treated by surgery or aspiration before entering the cohort study. NS, not stated.

To evaluate the sensitivity of the serological tests, the first available serum from patients with CE was used. This excluded 33 patients who had undergone surgery or percutaneous treatment before the time of collection of the first sample available for this study, and included 220 patients with detectable CE cysts, from which 220 serum samples were used for ELISA (123 collected from patients who were not treated with drugs when the first serum was collected, and 97 collected after drug treatment). From these, only 165 serum samples had enough volume to be also tested in the two IC devices (92 collected from patients without previous drug treatment and 73 collected after drug treatment).

Patients in follow-up with at least two consecutive serum samples (n = 105, from which only 74 had enough serum volume to be also tested in IC) were divided in three groups according to their clinical management: (i) surgical or percutaneous treatment, (ii) drug treatment only and (iii) non-treated watch and wait patients with CE4 and CE5 cysts. All patients included in the follow-up study had liver CE. Patients who had undergone percutaneous treatment or surgery were divided into cured patients with stages that had progressed to inactive residual lesions after percutaneous treatment or after surgery (n = 17, 55 samples tested with ELISA and n = 12, 28 samples tested with IC) and non-cured patients showing relapses (n = 4, 13 samples tested with ELISA and n = 3, 9 samples tested with IC). Patients treated with albendazole were divided into two groups. The first group comprised patients whose US image changed from active or transitional stages to inactive stages (CE4 and CE5) in response to treatment (n = 18, 70 samples tested with ELISA and n = 13, 42 samples, tested with IC) over the follow-up period, with no sign of reactivation after having reached the CE4 stage. The second group comprised patients with poor response to treatment, in whom there was no permanent change from active or transitional stages to inactive stages (treatment failure) during the follow-up period (n = 46, 186 samples tested with ELISA and n = 31, 106 samples tested with IC).

Twenty patients without treatment having inactive cysts managed with the watch and wait approach were checked for US changes during the follow-up period. No patient showed US changes. This group was assessed for SI in ELISA (n = 20, 63 samples) or band intensity in IC changes (n = 15, 38 samples) during the follow-up period.

Additionally, 25 patients who underwent drug treatment reached inactive cyst stage (CE4) during follow-up, while 24 untreated patients did so spontaneously, showing inactive cysts at baseline.

**Fig 2:**
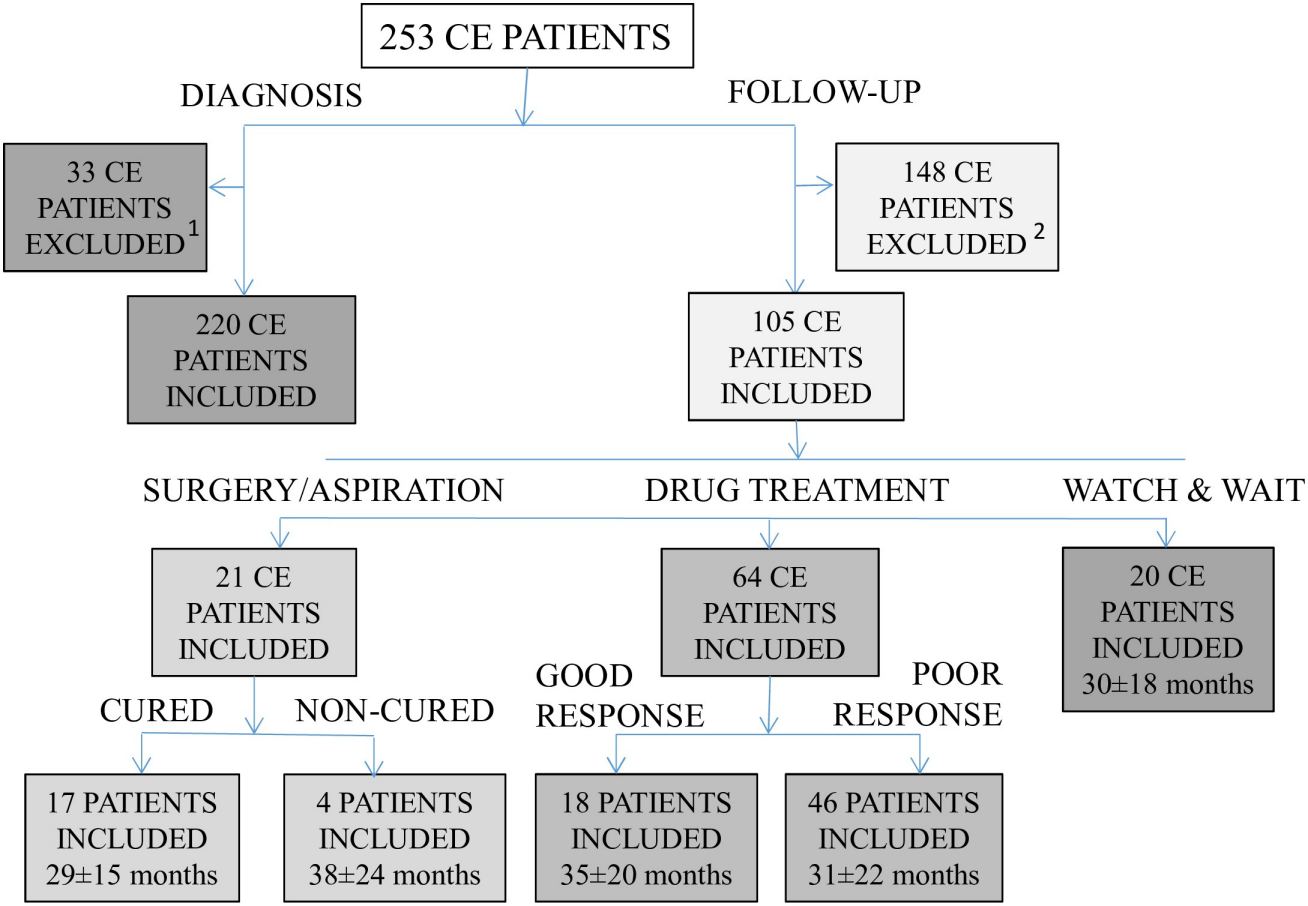
Flow chart showing the participants (cystic echinococcosis patients) in the study. ^1^Patients receiving surgery or percutaneous treatment before the collection of the first serum sample. ^2^Patients with only one serum sample.

A flow chart of participants, including the mean follow-up time with standard deviation (SD) and the number of samples collected for each of the above-mentioned groups is shown in Fig 2.

### Diagnostic sensitivity, specificity and cross-reactivity

Sensitivity, specificity and cross-reactivity for each test are shown in Table 2. From the highest to the lowest sensitivity, tests results were as follows: HF-ELISA (85.5%; 95% CI: 80.1–89.8%), VIRapid Hydatidosis (83.0%; 95% CI: 76.4–88.4%), IC containing 2B2t-HF (78.2%; 95% CI: 71.1–84.2%), B2t-ELISA and 2B2t-ELISA (51.8% both; 95% CI: 45.0–58.6%). When sensitivity was compared between samples collected before and after treatment, all tests performed better with serum samples collected after treatment, especially the two recombinant antigens in ELISA. Both recombinant antigens in ELISA showed a significantly lower sensitivity compared with the ELISA-HF and both IC tests.

**Table 2:** Sensitivity, specificity and cross-reactivity of the ELISA and IC tests.

		Hydatid fluid (ELISA)	B2t (ELISA)	2B2t (ELISA)	Virapid Hydatidosis (IC)	2B2t-HF (IC)
**SENSITIVITY (overall)**	**n/N**	188/220	114/220	114/220	137/165	129/165
	**%**	85.5%	51.8%^1,2^	51.8%^1,2^	83.0%	78.2%^1^
	**(95% CI)**	(80.1–89.8)	(45.0–58.6)	(45.0–58.6)	(76.4–88.4)	(71.1–84.2)
Before treatment	**n/N**	99/123	49/123	52/123	74/92	68/92
	**%**	80.5%	39.8%^1,2^	42.3%^1,2^	80.4%	73.9%^1,3^
	**(95% CI)**	(72.4–87.1)	(31.1–49.1)	(33.4–51.5)	(70.9–88.0)	(63.7–82.5)
After treatment	**n/N**	89/97	65/97	62/97	63/73	61/73
	**%**	91.8%	67.0%^1,2^	63.9%^1,2^	86.3%	83.6%
	**(95% CI)**	(84.4–96.4)	(56.7–76.2)	(53.5–73.4)	(76.2–93.2)	(73.0–91.2)
**SPECIFICITY**	**n/N**	64/82	68/77	81/82	28/30	30/30
	**%**	78.0%	88.3%^4^	98.8%^1^	93.3%	100%^1^
	**(95% CI)**	(67.6–86.4)	(79.0–94.5)	(93.4–100.0)	(77.9–99.2)	(88.4–100.0)
**CROSS-REACTIVITY**	**n/N**	22/42	4/42	7/42	27/42	13/42
	**%**	52.4%	9.5%^1,2^	16.7%^1,5^	64.3%	31.0%^3^
	**(95% CI)**	(36.4–68.0)	(2.7–22.6)	(7.0–31.4)	(48.0–78.4)	(17.6–47.1)

IC, immunochromatography; n, number of positive samples; N, total number of samples; %, percentage of positive samples. Statistically significant differences according to McNemar test conducted on paired samples are marked with ^1^ (between the marked tests and the HF-ELISA), ^2^ (between the marked test and both IC tests), ^3^ (between the two IC tests), ^4^ (between the marked test and the 2B2t-HF IC test), and ^5^ (between the marked test and the Virapid Hidatidosis IC test).

Specificity as assessed with sera from healthy donors, from the highest to the lowest, was as follows: IC containing 2B2t-HF (100%; 95% CI: 88.4–100%), 2B2t-ELISA (98.8%; 95% CI: 93.4–100%), VIRapid Hydatidosis (93.3%; 95% CI: 77.9–99.2%), B2t-ELISA (88.3%; 95% CI: 79.0–94.5%) and HF-ELISA (78%; 95% CI: 67.6–86.4%) (Table 2). Cross-reactivity of the different tests checked against samples from patients with AE was very high for the HF both in the VIRapid Hydatidosis IC (64.3%; 95% CI: 48.0–78.4%) and ELISA (52.4%; 95% CI: 36.4–68.0), and moderate to low for the recombinant antigens in IC (31.0%; 95% CI: 17.6–47.1%) and ELISA (16.7%; 95% CI: 7.0–31.4% for 2B2t and 9.5%; 95% CI: 2.7–22.6% for B2t). Statistically significant differences in specificity were found between the ELISA-HF and both the 2B2t-ELISA and 2B2t-HF IC test, and between the latter and the B2t-ELISA test. Differences in cross-reactivity were statistically significant between both recombinant antigens in ELISA and the HF-based ELISA and VIRapid Hydatidosis IC test, between B2t-ELISA and 2B2t-HF IC, and between IC tests.

### Variables influencing serology results

The analysis of the association between clinical variables and sensitivity of the different ELISA and IC tests is presented in Table 3. Independently of the other clinical characteristics, cyst stage was found to influence sensitivity of all serological tests. Among patients harboring active or transitional cysts, the highest sensitivity was obtained in HF-ELISA, with the exception of patients with CE3b cysts, among whom the 2B2t-HF IC showed the highest sensitivity. Sensitivity of the two recombinant antigens in ELISA was the lowest for all cyst stages, ranging from 84.6% (2B2t for CE1 cysts) to 64.8% (B2t for CE3b cysts) among patients harboring active cysts, and drastically dropping among those with inactive cysts, especially CE5 cysts (5.1% and 2.6% for B2t and 2B2t, respectively).

**Table 3:** Sensitivity of the serological tests according to different clinical characteristics.

	Hydatid fluid (ELISA)	B2t (ELISA)	2B2t (ELISA)	Virapid Hydatidosis	2B2t-HF (IC)
**Serum collection**	P = 0.116^1^	**P = 0.001**^1^	**P = 0.013**^1^	P = 0.920^1^	P = 0.584^1^
**Before treatment**; n (%)	99 (80.5)	49 (39.8)	52 (42.3)	74 (80.4)	68 (73.9)
**After treatment**; n (%)	89 (91.8)	65 (67.0)	62 (63.9)	63 (86.3)	61 (83.6)
**Number of cysts**	P = 0.484^1^	P = 0.447^1^	P = 0.223^1^	P = 0.825^1^	P = 0.415^1^
**1**; n (%)	117 (82.4)	66 (46.5)	65 (45.8)	85 (80.2)	79 (74.5)
**> 1**; n (%)	71 (91.0)	48 (61.5)	49 (62.8)	52 (88.1)	50 (84.7)
**Cyst stage**	**P = 0.025**^1^	**P < 0.001**^1^	**P < 0.001**^1^	**P = 0.035**^1^	**P = 0.002**^1^
**CE1**; n (%)	13 (100.0)	10 (76.9)	11 (84.6)	10 (90.9)	10 (90.9)
**CE2**; n (%)	20 (100.0)	15 (75.0)	15 (75.0)	18 (94.7)	17 (89.5)
**CE3a**; n (%)	28 (100.0)	22 (78.6)	23 (82.1)	22 (100.0)	21 (95.5)
**CE3b**; n (%)	66 (93.0)	46 (64.8)	47 (66.2)	48 (92.3)	49 (94.2)
**CE4**; n (%)	37 (75.5)	19 (38.8)	17 (34.7)	29 (72.5)	25 (62.5)
**CE5**; n (%)	24 (61.5)	2 (5.1)	1 (2.6)	10 (47.6)	7 (33.3)
**Cyst size**	P = 0.112^1^	P = 0.605^1^	P = 0.105^1^	P = 0.119^1^	**P = 0.014**^1^
**Small**; n (%)	66 (78.6)	38 (45.2)	34 (40.5)	48 (72.7)	42 (63.6)
**Medium**; n (%)	93 (88.6)	56 (53.3)	59 (56.2)	66 (90.4)	64 (87.7)
**Big**; n (%)	15 (100.0)	10 (66.7)	10 (66.7)	11 (91.7)	12 (100.0)
**Cyst location**	**P = 0.017**^1^	P = 0.078^1^	P = 0.362^1^	P = NC^1^	P = 0.NC^1^
**Liver**; n (%)	177 (87.2)	107 (52.7)	106 (52.2)	130 (82.8)	122 (77.7)
**Other organs**; n (%)	10 (62.5)	6 (37.5)	7 (43.8)	6 (85.7)	6 (85.7)

^1^P-values estimated through multivariable logistic regression accounting for the potential confounding due to all variables presented in the table. The multivariable analysis was conducted on 203 and 150 patients with data available for all variables included into the models for ELISA and IC, respectively. Statistically significant differences are highlighted in bold; NC, not calculable because of empty cells in multivariable analysis.

Sensitivity of the HF-based ELISA was significantly influenced by the location of cysts, with a higher sensitivity observed among samples of patients with cysts located in the liver compared with those of patients with cysts located in other organs. The time point of serum collection showed to influence the sensitivity of ELISA based on the recombinant antigens, which was found to be significantly higher among samples collected after treatment. Finally, sensitivity of the IC containing the 2B2t recombinant antigen was found to significantly increase with cyst size.

### Tests performances for patients in follow-up

**Fig 3:**
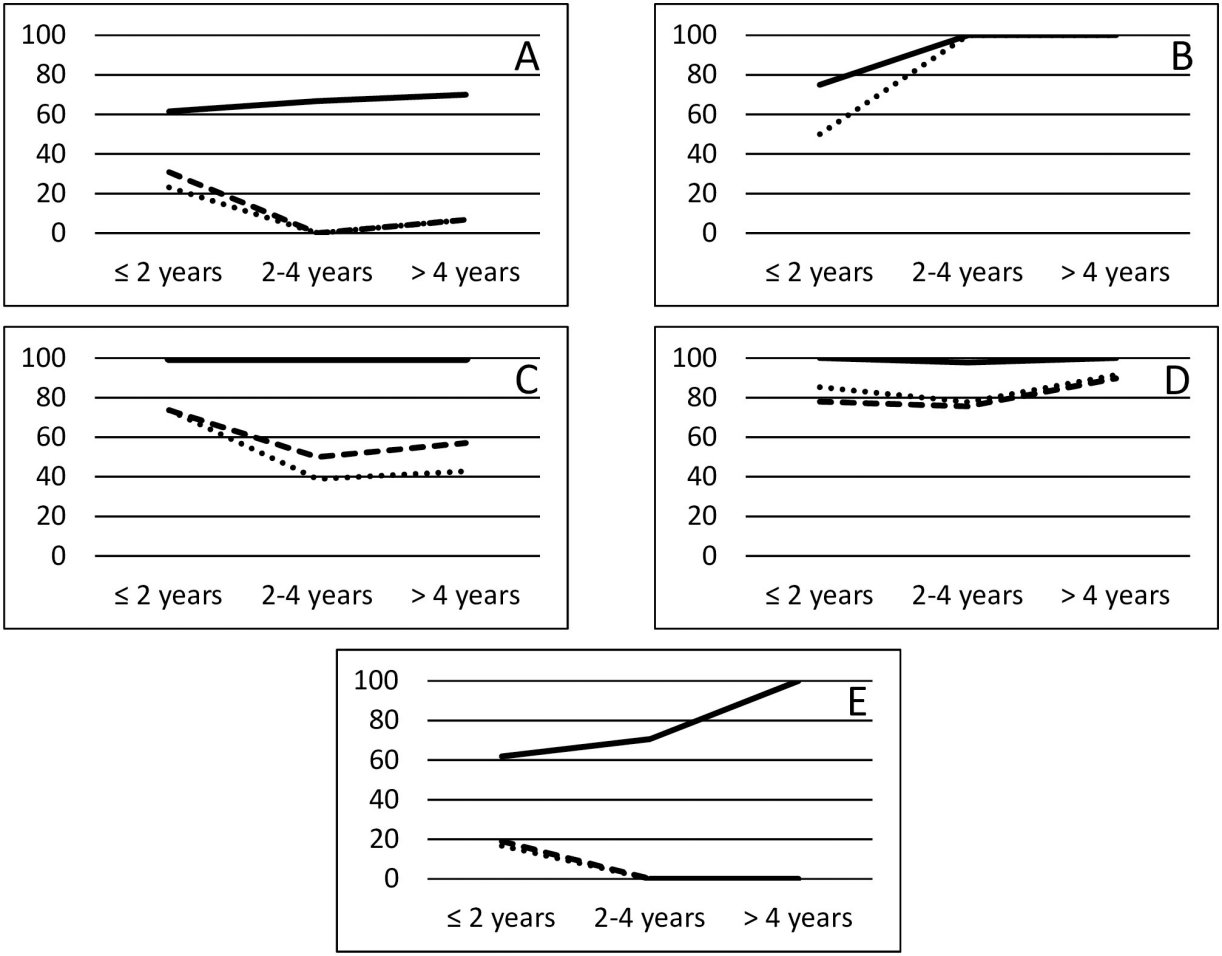
Percentage of positive sera (Y axis) against hydatid fluid (black line), B2t (dashed line) and 2B2t (dotted line) recombinant antigens in ELISA. The percentage was calculated at different time intervals after treatment (years, X axis) for patients subjected to surgery or aspiration and cured (A) or non-cured (B), subjected to drug treatment and with good response (C) or poor response (D), and at different time intervals for patients in watch and wait (E). In (B), the trend line is coincident for the B2t and 2B2t antigens.

The percentages of positive samples in ELISA tests over time after surgery or percutaneous treatment among patients cured and not cured are shown in (Fig 3A and 3B). Overall, the percentage of positive samples in cured patients decreased over time when using recombinant antigens but not HF (Fig 3A), while this percentage increased over time and remained stable at 100% in non-cured patients using all antigens (Fig 3B). Evolution with time of the percentage of positive samples in HF-ELISA showed a constant trend, with percentage at about 100%, among all patients who underwent drug treatment, regardless of their response to therapy (Fig 3C and 3D). Among patients with both good or poor response to drug treatment, a drop in the percentage of samples tested positive using recombinant antigens was observed in the second time period assessed (2 to 4 years since the start of treatment), with an increase in the last time period (> 4 years since the start of treatment). Among untreated patients, the percentage of positive samples in HF-ELISA was over 60% for the first evaluated period (0 to 2 years from baseline), increasing to 100% after 4 years of follow-up. On the contrary, positivity rate in ELISA based on recombinant antigens was very low (< 20%) at the beginning of the studied period and decreased over time to reach 0% after 4 years (Fig 3E).

Progression to negativity by treatment outcome (cured *vs*. non-cured patients) among patients who underwent surgical intervention or percutaneous treatment and classified as positive at baseline is shown in Table 4 separately for each test. The B2t-ELISA is the only test showing a statistically significant difference in the incidence rate of negativization between cured and non-cured patients. This is also the antigen showing the best incidence of negativization in cured patients (13.04 per 100 PM), followed by the 2B2t in ELISA (9.52 per 100 PM), the HF-based IC strip (1.16 per 100 PM) and the HF-ELISA (1.01 per 100 PM). None of the patients with a negative outcome (non-cured) was tested negative with any test during the follow-up.

**Table 4:** Progression to negativity by treatment outcome (cured *vs*. non-cured patients) among patients who underwent surgical intervention or percutaneous treatment and classified as positive at baseline (time at first test available).

TEST	no. negative	Person-months follow-up (PM)	Neg. rate *100 PM (95% CI)	P-value^1^
**Hydatid fluid (ELISA)**				0.302
Cured (N = 13)	3	296	1.01 (0.33–3.14)	
Not cured (N = 3)	0	138	0.00	
**B2t (ELISA)**				**0.042**
Cured (N = 4)	3	23	13.04 (4.21–40.44)	
Not cured (N = 3)	0	138	0.00	
**2B2t (ELISA)**				0.059
Cured (N = 3)	2	21	9.52 (2.38–38.08)	
Not cured (N = 3)	0	138	0.00	
**Virapid Hydatidosis**				0.513
Cured (N = 8)	1	86	1.16 (0.16–8.21)	
Not cured (N = 3)	0	101	0.00	
**2B2t (IC)**				NC
Cured (N = 6)	0	78	0.00	
Not cured (N = 3)	0	101	0.00	

^1^ Log-rank test. Significant P values are marked in bold.

“no. negative”: number of patients who become negative (SI < 50) during the follow-up, among the N patients who were positive (SI ≥ 50) at baseline (first test available). “Person-months follow-up (PM)”: for each group, total number of months elapsed from baseline to negativization (patients who became negative during follow-up), or from baseline to last test available (patients who remained positive during follow-up). “Neg. rate*100 PM”: incidence density rate of negativization (no. negative/PM*100). NC, not calculable.

Incidence of negativization in patients who underwent drug treatment and classified as positive at baseline did not show any statistically significant difference between patients with good and poor response to treatment (Table 5).

**Table 5:** Progression to negativity by treatment response (good *vs*. poor) among patients who underwent drug treatment and classified as positive at baseline (time at first test available).

TEST	no. negative	Person-months follow-up (PM)	Neg. rate *100 PM (95% CI)	P-value^1^
**Hydatid fluid (ELISA)**				NC
Good (N = 18)	0	625	0.00	
Poor (N = 45)	0	1417	0.00	
**B2t (ELISA)**				0.389
Good (N = 14)	6	391	1.53 (0.69–3.42)	
Poor (N = 35)	9	966	0.93 (0.48–1.79)	
**2B2t (ELISA)**				0.123
Good (N = 14)	6	387	1.55 (0.70–3.45)	
Poor (N = 35)	6	1038	0.58 (0.26–1.29)	
**Virapid Hydatidosis**				NC
Good (N = 12)	0	332	0.00	
Poor (N = 29)	0	705	0.00	
**2B2t (IC)**				NC
Good (N = 12)	0	332	0.00	
Poor (N = 30)	0	715	0.00	

^1^ Log-rank test. “no. negative”: number of patients who become negative (SI < 50) during the follow-up, among the N patients who were positive (SI ≥ 50) at baseline (first test available). “Person-months follow-up (PM)”: for each group, total number of months elapsed from baseline to negativization (patients who became negative during follow-up), or from baseline to last test available (patients who remained positive during follow-up). “Neg. rate*100 PM”: incidence density rate of negativization (no. negative/PM*100). NC, not calculable.

Moreover, progression to negativity in patients who were positive at baseline did not significantly differ between patients under drug treatment with good response and patients followed with the watch and wait approach (Table 6). However, the median SI in drug-treated patients at the time inactive cysts (CE4) were detected was significantly higher compared to median SI in watch-wait patients in all ELISA tests (Table 7).

**Table 6:** Progression to negativity by treatment group (drug^1^ and watch-wait) among patients who were positive at baseline (time at first test available).

TEST	no. negative	Person-months follow-up (PM)	Neg. rate *100 PM (95% CI)	P-value^2^
**Hydatid fluid (ELISA)**				0.072
Drug^1^ (N = 18)	0	625	0.00	
Watch-wait (N = 13)	2	366	0.55 (0.14–2.18)	
**B2t (ELISA)**				0.155
Drug^1^ (N = 14)	6	391	1.53 (0.69–3.42)	
Watch-wait (N = 3)	1	39	2.56 (0.36–18.20)	
**2B2t (ELISA)**				0.062
Drug^1^ (N = 14)	6	387	1.55 (0.70–3.45)	
Watch-wait (N = 3)	2	43	4.65 (1.16–18.60)	
**Virapid Hydatidosis**				0.210
Surgery (N = 11)	1	187	0.53 (0.08–3.79)	
Drug^1^ (N = 12)	0	332	0.00	
Watch-wait (N = 9)	1	180	0.56 (0.08–3.94)	
**2B2t (IC)**				0.176
Drug^1^ (N = 12)	0	332	0.00	
Watch-wait (N = 8)	1	155	0.65 (0.09–4.58)	

^1^ Drug-treated patients with good response (reaching CE4 or CE5 cyst stage during follow-up);

^2^ Log-rank test. “no. negative”: number of patients who become negative (SI < 50) during the follow-up, among the N patients who were positive (SI ≥ 50) at baseline (first test available). “Person-months follow-up (PM)”: for each group, total number of months elapsed from baseline to negativization (patients who became negative during follow-up), or from baseline to last test available (patients who remained positive during follow-up). “Neg. rate*100 PM”: incidence density rate of negativization (no. negative/PM*100).

**Table 7:** Comparison of the SI of patients with CE4 cysts in non-treated patients and under drug treatment against hydatid fluid, B2t and 2B2t in ELISA.

	PATIENTS UNDER DRUG TREATMENT^1^ (N = 25)	NON-TREATED PATIENTS (N = 24)	p-value^2^
	Median (IQR)		
**Hydatid fluid**	283 (252–314)	88 (19–263)	**< 0.001**
**B2t**	73 (10–139)	1 (-14–25)	**< 0.001**
**2B2t**	83 (20–169)	3(-6–47)	**0.002**

^1^ only drug-treated patients who reached CE4 stage;

^2^ Wilcoxon rank-sum test. Significant P values are marked in bold. IQR, interquartile range.

## Discussion

The recombinant antigens B2t and 2B2t [9, 12] were tested in ELISA to compare their sensitivity with that of HF using an extended number of samples. Additionally, the 2B2t antigen was included in an IC test and compared with the HF (VIRapid Hydatidosis) in the same test. Similar to our previous results, recombinant antigens showed lower sensitivity compared to HF in ELISA, and although the recombinant 2B2t antigen showed enhanced sensitivity in the IC test, this was still lower than the sensitivity of the HF. A deeper analysis of the different clinical variables that could account for the low sensitivity of the recombinant antigens in ELISA showed that these were influenced by the cyst stage and the timing of serum collection (before and after drug treatment). Patients with inactive cysts were clearly testing more frequently negative with the two recombinant antigens than patients with active or transitional cysts. These differences were also statistically significant for the HF-ELISA and for the two IC tests.

Similar results were obtained by Pagnozzi et al. [38] using a purified Ag5 from HF in ELISA and by Yang et al. [39] using AgB1. Tamarozzi et al. [22] also detected a statistically significant difference between OD values of active and inactive cyst groups against the recombinant antigen B1 in ELISA. In a recent study, variation of the immune-proteome profile of HF along the cyst progression has been shown [25], with patients having CE2 and CE3 stages exhibiting strong antibody responses against diverse AgB and Ag5 proteoforms, while sera from patients with CE1, CE4, and CE5 stages mainly reacting to Ag5 and cathepsin B but not against AgB proteins. This has been attributed to changes in the antigenic composition of cysts depending on cyst stages, e.g., the AgB2 antigen expression is reduced in degenerating and inactive cysts [40]. This supports empirically the finding that the different antigenic composition of the various cyst stages could largely influence the antibody reactivity found in patients with active, transitional or inactive cysts against specific antigens, including AgB2 antigen. In this respect, sensitivity of serological tests could be enhanced combining several recombinant antigens, as suggested by Jiang et al. [23], who found a differential response of patients with CE against different recombinant antigen B isoforms. Additionally, it should be mentioned that AgB genes present high polymorphism and variable transcription profiles in different *E*. *granulosus* genotypes (rev. in [4]). This could also influence the immune response against the corresponding native and recombinant antigens.

The second variable statistically influencing the positivity in the ELISA containing any of the two recombinant antigens tested here was the drug treatment. Albendazole treatment results in a significant increased number of patients testing positive against B2t and 2B2t. Drug treatment similarly affects the serological results obtained with the recombinant antigen B1 [6, 22]. These authors have suggested that drug treatment could result in the spillage of antigens from damaged cysts and in the increase of antibody responses, which seems to be especially true for defined antigens [22], at least during one year post-treatment [6].

Sensitivity of the ELISA test based on the recombinant antigen B2t reported in previous works contrasts with what found here. Hernandez-Gonzalez et al. [12] reported a sensitivity of 91.2% for B2t antigen in ELISA, similar to that reported for HF in the same publication. Nevertheless, the majority of clinical data of the 102 CE patients whose sera were included in that publication were lacking, including cyst stage, thus comparison of those results with the present work is not feasible. Later, an extended panel of sera from patients with CE whose clinical data were available was tested against both recombinant antigens B2t and 2B2t in ELISA [9]. Sensitivity reported in that study was 79% for B2t and 87.6% for 2B2t. Although closer to results of the present study, it is noteworthy that of the 186 sera tested, only 2 were from patients with CE4 cysts, and none from patients with CE5 cysts. To further stress the importance of the distribution of cyst stages in the panel of samples used for accuracy studies, the logistic regression analysis showed that only cyst number per patient influenced the outcome of tests based on either recombinant antigen, thus the reliability of the influence of the cyst stage in the performance of these antigens was biased in that study. Indeed, the mean value of sensitivity for active and transitional cysts in our study was 73.8% for B2t and 76.9% for 2B2t, close to that found by Hernandez-Gonzalez et al. [9] for the same antigens mainly challenged with sera from patients with active and transitional cysts. Additionally, other forms of the recombinant AgB1 and AgB2 have been tested by different authors, showing in general higher sensitivity than the detected here (rev. in [4]). Majority of these previous studies cannot be compared with the present study, due to the lack of clinical data in most of the studies, although differences could be also attributed, at least in part, to the antigenic differences among different versions of the same recombinant antigen.

When the recombinant antigen 2B2t was used in the IC test combined with HF in the conjugate, the sensitivity was higher than that found for the same recombinant antigen in ELISA. In particular, increased sensitivity was found for the detection of inactive cysts, similar to what observed using the IC test containing only HF (VIRapid Hydatidosis), but with higher specificity and less cross-reactivity with sera from patients with AE. These results suggest the use of recombinant antigens in combination with HF in easy-to-use diagnostic tests to improve the performance of tests based only on HF.

One of the many contentious points in the clinical management of patients with CE is the usefulness of serology for the follow-up of treated patients. Antibodies against HF persist after cure, and although specific antibody isotypes and sub-isotypes against HF, including IgE, IgM, IgG2 and IgG4, have been suggested to be of use for follow-up by several authors, the demonstration of usefulness of serology has been hampered by the low number of tested samples in published work and the lack of these antibodies in a percentage of patients with CE (rev. in [4, 5]). Similarly, the use of a number of recombinant or purified native antigens has been investigated for the follow-up of treated patients. For example, a drop in specific antibody titers after successful surgical treatment for CE has been reported against the recombinant AgB, AgB2t, AgP29, and HSP20 [12, 39, 41, 42, 43]. Published work shows that cytokine detection seems not to be more useful than antibody detection for primary diagnosis of CE patients (rev. in Siles-Lucas et al., 2017). Detection of specific cytokines and their fluctuation (e.g., IL-4; [44, 45]) could have potential in the definition of cyst vitality (viability) and follow-up of treated patients, with limitations regarding test sensitivity and available facilities. Additionally, extended cohorts of patients should be tested before drawing conclusions about the usefulness of cytokine detection in the follow-up of CE patients. Here, our results also show a decrease in the number of positive patients against the recombinant antigen-based ELISA in cured patients after surgery or percutaneous treatment, in comparison with non-cured patients, especially against the B2t antigen. Taken together, these results suggest that serology performed using defined recombinant antigens could be useful for the follow-up of surgically and percutaneously treated patients. However, this approach is only possible when patients show positive serology against the target antigens, a condition unfortunately found only in a variable percentage of subjects (e.g. for P29 and 2B2t; [42, 43]). This was also true for the recombinant antigens B2t and 2B2t in the present study, and although a decline in specific antibodies can be detected in cured patients after intervention, the number of patients testing positive at baseline was very low. This is related with one of the limitations of the present study, because although the number of patients in this clinical management modality was preliminary acceptable for statistical analysis, those showing positive serology at the time of first test available were limited. Nevertheless, sample size was enough to suggest the uselessness of HF and the apparent uselessness of the IC test for the follow-up of patients after surgery or aspiration treatment, due to persistence of positivity against them for prolonged time regardless of treatment outcome. Very similar conclusions can be extracted from our results regarding the usefulness of serology based in HF for the follow-up of drug-treated patients. Contrasting, recombinant antigens in ELISA showed here a higher number of positive patients at baseline for drug treated patients and results suggest that progression to negativity is faster in patients with good response to treatment compared with patients with poor response, although differences were not statistically significant.

Thus, the low reactivity of B2t and 2B2t recombinant antigens against sera from patients with inactive cysts, similar to what reported for other recombinant antigens (e.g. HSP60 and B1; [39, 46]) could be also of use for the follow-up of patients treated medically. In these patients, US evolution from active or transitional to inactive stage may be achieved, but a proportion of cysts, although inactive on imaging, remain biologically viable, as reactivation is observed over time. This is best shown by the behavior of CE3b cysts that almost invariably reactivate, after an initial solidification, after the end of albendazole treatment [18, 47].

Patients analyzed here with spontaneously inactivated CE4/CE5 cysts without treatment (in watch and wait) showed no changes in cyst stage along the follow-up period, in accordance with what observed in previous studies [27, 47, 48]. However, a decline of specific antibodies along the follow-up could be detected. This shows, as expected, that a period of time has to elapse between the inactivation of the cyst and the loss of circulating antibodies against defined antigens.

Further follow-up studies using B2t and 2B2t-based serology on a cohort of patients undergoing inactivation and reactivation, not available in this cohort, would therefore be of interest to assess if tests based on these antigens can indicate evolution of cysts before morphological features change on imaging, thus reducing patient follow-up. This would be absolutely useful especially in resource poor settings where expertise and availability of portable US are scant, where the reliability of such result, especially if obtainable using a rapid IC test, could save transportation time and costs for patient. In the present study, antibody levels were significantly higher in patients with CE4 cysts that have evolved from active or transitional stages after drug treatment than in patients reaching the CE4 stage spontaneously. Whether the higher number of positive, drug-treated patients with CE4 cysts compared with patients with “spontaneous” CE4 cysts is due to the incomplete loss of viability of CE4 cysts after drug treatment, as is highly likely, should be further investigated.

In summary, sensitivity of serodiagnostic tests based in ELISA containing only derivatives of AgB2 in their recombinant form is substantially lower compared to tests based on HF, especially for the detection of patients with CE harboring inactive cysts. However, this lower sensitivity for inactive lesions could be exploitable for the follow-up of patients whose serology is positive against the recombinant B antigens at the beginning of observation. Nonetheless, specificity and cross-reactivity performance of the recombinant antigens was much better than the performance of the HF, which indicates the use of these antigens especially in patients showing images of lesions similar to CE. A further encouraging result is that the combination of recombinant antigens derived from AgB2 with HF in easy-to-use devices (IC) has improved sensitivity compared to tests based only on recombinant antigens in ELISA, while offering higher specificity and less cross-reactivity than similar IC devices containing only HF. The IC test containing the 2B2t recombinant antigen was also tested for its usefulness in the follow-up of patients with CE for the above-mentioned groups. No statistically significant differences were found when comparing any of the patients’ groups, thus showing no applicability in the follow-up of patients with CE after treatment.

## Supporting information

S1 Checklist STROBE Checklist. (DOC)
