# Supplementary material for: Evaluation of the recombinant antigens B2t and 2B2t, compared with hydatid fluid, in IgG-ELISA and immunostrips for the diagnosis and follow up of CE patients
Source: PLoS Negl Trop Dis. 2018 Sep 6;12(9):e0006741. doi: 10.1371/journal.pntd.0006741 (PMC6143278; doi:10.1371/journal.pntd.0006741)
Supplement: S1 Checklist — (DOC) [file pntd.0006741.s001.doc]

STROBE Statement—Checklist of items that should be included in reports of ***cohort studies***

|  | Item No | Recommendation |
| --- | --- | --- |
| **Title and abstract** | 1 | (*a*) Indicate the study’s design with a commonly used term in the title or the abstract  ABSTRACT, LINE 12 |
|  |  | (*b*) Provide in the abstract an informative and balanced summary of what was done and what was found  ABSTRACT, LINE 10 TO 28 |
| Introduction | | |
| Background/rationale | 2 | Explain the scientific background and rationale for the investigation being reported  INTRODUCTION, PARAGRAPH 1 TO 3 |
| Objectives | 3 | State specific objectives, including any prespecified hypotheses  INTRODUCTION, PARAGRAPHS 4 TO 6 |
| Methods | | |
| Study design | 4 | Present key elements of study design early in the paper  METHODS, FIRST PARAGRAPH, UNDER *Study design and samples* SECTION |
| Setting | 5 | Describe the setting, locations, and relevant dates, including periods of recruitment, exposure, follow-up, and data collection  METHODS, PARAGRAPHS 2 AND 3, UNDER *Study design and samples* SECTION |
| Participants | 6 | (*a*) Give the eligibility criteria, and the sources and methods of selection of participants. Describe methods of follow-up  METHODS, FIRST PARAGRAPH, UNDER *Study design and samples* SECTION |
|  |  | (*b*) For matched studies, give matching criteria and number of exposed and unexposed  NOT APPLICABLE |
| Variables | 7 | Clearly define all outcomes, exposures, predictors, potential confounders, and effect modifiers. Give diagnostic criteria, if applicable  METHODS, PARAGRAPHS 2 AND 3, UNDER *Study design and samples* SECTION |
| Data sources/ measurement | 8* | For each variable of interest, give sources of data and details of methods of assessment (measurement). Describe comparability of assessment methods if there is more than one group  METHODS, ALL PARAGRAPHS UNDER *Test methods* SECTION |
| Bias | 9 | Describe any efforts to address potential sources of bias  METHODS, PARAGRAPH 2 UNDER *Test methods* SECTION |
| Study size | 10 | Explain how the study size was arrived at  METHODS, PARAGRAPH 1, UNDER *Study design and samples* SECTION |
| Quantitative variables | 11 | Explain how quantitative variables were handled in the analyses. If applicable, describe which groupings were chosen and why  METHODS, ALL PARAGRAPHS UNDER *Statistical analysis* SECTION |
| Statistical methods | 12 | (*a*) Describe all statistical methods, including those used to control for confounding  METHODS, ALL PARAGRAPHS UNDER *Statistical analysis* SECTION |
|  |  | (*b*) Describe any methods used to examine subgroups and interactions  METHODS, ALL PARAGRAPHS UNDER *Statistical analysis* SECTION |
|  |  | (*c*) Explain how missing data were addressed  NO MISSING DATA |
|  |  | (*d*) If applicable, explain how loss to follow-up was addressed  NOT APPLICABLE |
|  |  | (*e*) Describe any sensitivity analyses  METHODS, PARAGRAPHS 2 AND 3 UNDER *Statistical analysis* SECTION |
| Results | | |
| Participants | 13* | (a) Report numbers of individuals at each stage of study—eg numbers potentially eligible, examined for eligibility, confirmed eligible, included in the study, completing follow-up, and analysed  RESULTS, PARAGRAPHS UNDER *Samples* SECTION |
|  |  | (b) Give reasons for non-participation at each stage  RESULTS, PARAGRAPHS UNDER *Samples* SECTION |
|  |  | (c) Consider use of a flow diagram  RESULTS, LAST PARAGRAPH UNDER *Samples* SECTION (FIG. 2) |
| Descriptive data | 14* | (a) Give characteristics of study participants (eg demographic, clinical, social) and information on exposures and potential confounders  RESULTS, FIRTS PARAGRAPH UNDER *Samples* SECTION (TABLE 1) |
|  |  | (b) Indicate number of participants with missing data for each variable of interest  RESULTS, PARAGRAPHS UNDER *Samples* SECTION |
|  |  | (c) Summarise follow-up time (eg, average and total amount)  RESULTS, LAST PARAGRAPH UNDER *Samples* SECTION (FIG. 2) |
| Outcome data | 15* | Report numbers of outcome events or summary measures over time  DONE |
| Main results | 16 | (a) Give unadjusted estimates and, if applicable, confounder-adjusted estimates and their precision (eg, 95% confidence interval). Make clear which confounders were adjusted for and why they were included  RESULTS, PARAGRAPHS 1 AND 2, UNDER *Diagnostic sensitivity, specificity and cross-reactivity* SECTION |
|  |  | (*b*) Report category boundaries when continuous variables were categorized  NOT APPLICABLE |
|  |  | (*c*) If relevant, consider translating estimates of relative risk into absolute risk for a meaningful time period  NOT RELEVANT |
| Other analyses | 17 | Report other analyses done—eg analyses of subgroups and interactions, and sensitivity analyses  RESULTS, PARAGRAPHS UNDER *Variables influencing serology results* SECTION |
| Discussion | | |
| Key results | 18 | Summarise key results with reference to study objectives  DISCUSSION, PARAGRAPHS 1 AND 6 |
| Limitations | 19 | Discuss limitations of the study, taking into account sources of potential bias or imprecision. Discuss both direction and magnitude of any potential bias  DISCUSSION, PARAGRAPHS 6 AND 9 |
| Interpretation | 20 | Give a cautious overall interpretation of results considering objectives, limitations, multiplicity of analyses, results from similar studies, and other relevant evidence  DISCUSSION, LAST PARAGRAPH |
| Generalisability | 21 | Discuss the generalisability (external validity) of the study results  DISCUSSION, LAST PARAGRAPH |
| Other information | | |
| Funding | 22 | Give the source of funding and the role of the funders for the present study and, if applicable, for the original study on which the present article is based  INFORMATION INCLUDED IN THE ONLINE EDITORIAL SYSTEM |

*Give information separately for exposed and unexposed groups.

**Note:** An Explanation and Elaboration article discusses each checklist item and gives methodological background and published examples of transparent reporting. The STROBE checklist is best used in conjunction with this article (freely available on the Web sites of PLoS Medicine at http://www.plosmedicine.org/, Annals of Internal Medicine at http://www.annals.org/, and Epidemiology at http://www.epidem.com/). Information on the STROBE Initiative is available at http://www.strobe-statement.org.
